# Direct reprogramming of fibroblasts into spiral ganglion neurons by defined transcription factors

**DOI:** 10.1111/cpr.13775

**Published:** 2024-11-17

**Authors:** Yuhang Huang, Zhen Chen, Jiang Chen, Jingyue Liu, Cui Qiu, Qing Liu, Linqing Zhang, Guang‐Jie Zhu, Xiaofeng Ma, Shuohao Sun, Yun Stone Shi, Guoqiang Wan

**Affiliations:** ^1^ MOE Key Laboratory of Model Animal for Disease Study, Department of Otolaryngology Head and Neck Surgery, Jiangsu Provincial Key Medical Discipline (Laboratory), The Affiliated Drum Tower Hospital of Medical School and the Model Animal Research Center of Medical School Nanjing University Nanjing China; ^2^ State Key Laboratory of Pharmaceutical Biotechnology, Jiangsu Key Laboratory of Molecular Medicine, National Resource Center for Mutant Mice of China Nanjing University Nanjing China; ^3^ Department of Neurology, The Affiliated Drum Tower Hospital of Medical School and Institute of Translational Medicine for Brain Critical Diseases Nanjing University Nanjing China; ^4^ National Institute of Biological Sciences Beijing China; ^5^ Tsinghua Institute of Multidisciplinary Biomedical Research Tsinghua University Beijing China; ^6^ Research Institute of Otolaryngology Nanjing China; ^7^ Guangdong Institute of Intelligence Science and Technology Zhuhai China

## Abstract

Degeneration of the cochlear spiral ganglion neurons (SGNs) is one of the major causes of sensorineural hearing loss and significantly impacts the outcomes of cochlear implantation. Functional regeneration of SGNs holds great promise for treating sensorineural hearing loss. In this study, we systematically screened 33 transcriptional regulators implicated in neuronal and SGN fate. Using gene expression array and principal component analyses, we identified a sequential combination of Ascl1, Pou4f1 and Myt1l (APM) in promoting functional reprogramming of SGNs. The neurons induced by APM expressed mature neuronal and SGN lineage‐specific markers, displayed mature SGN‐like electrophysiological characteristics and exhibited single‐cell transcriptomes resembling the endogenous SGNs. Thus, transcription factors APM may serve as novel candidates for direct reprogramming of SGNs and hearing recovery due to SGN damages.

## Graphical abstract

A combination of Ascl1, Pou4f1 and Myt1l was identified to promote functional reprogramming of cochlear spiral ganglion neurons based on biochemical, electrophysiological and single‐cell transcriptomic analyses.

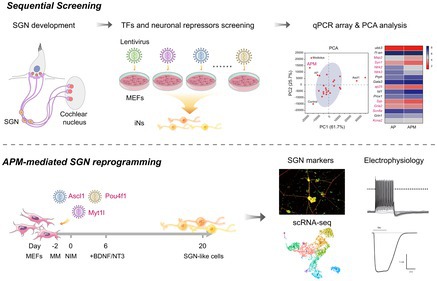

## 1 INTRODUCTION

In the cochlea of mammals, spiral ganglion neurons (SGNs) transmit auditory signals to the central circuits responsible for processing sound. SGNs are glutamatergic bipolar sensory neurons, which can be characterized as Type I (90%–95%) and Type II (5%–10%) and with neurites extending towards the bases and sides of the hair cells (HCs) to receive synaptic input. SGNs transmit auditory signals from HCs to the central auditory pathway through the generation of action potentials (APs). SGNs play a critical role in normal auditory function and communication. Sensorineural hearing loss results from irreversible damage to HCs and SGNs in mammals.^1^ Due to the inability of SGNs to regenerate in mammals, any damage, like ototoxicity, noise exposure and ageing, can lead to degeneration of SGN nerve terminals or cell bodies and result in irreparable hearing loss.^2^, ^3^, ^4^, ^5^, ^6^, ^7^, ^8^ The efficacy of hearing aids and cochlear implants depends on the presence and health of functional SGNs.^9^ The potential replacement or regeneration of SGNs could offer hope for patients with severe SGN damage and improve outcomes for individuals utilizing hearing devices.^10^

There are several important considerations for in vivo SGN reprogramming and regeneration, including progenitor cell identification, reprogramming approaches and functional recovery assessment.^11^, ^12^, ^13^ Cochlear resident cells, such as fibroblasts and glia cells, represent promising progenitor candidates for SGN reprogramming.^14^ Developmental pathways may also inform the selection of transcription factors (TFs) and small molecule combinations for effective SGN fate reprogramming.^15^, ^16^, ^17^, ^18^ Transcriptomic and electrophysiological analyses provide crucial metrics for evaluating the functions of the induced neurons (iNs) in vitro.^11^ However, successful hearing restoration ultimately requires reestablishing functional synaptic connections in vivo, presenting a major challenge in SGN regeneration.^8^, ^19^

Multiple signalling pathways and TFs coordinately regulate the development, differentiation and function of SGNs. During early development, the Notch signalling pathway participates in neuroblast delamination through lateral inhibition.^20^ In addition, IGF and Shh, Wnt and other signalling pathways promote the expansion and survival of neuroblasts.^21^, ^22^, ^23^ Neurotrophic factors such as brain‐derived neurotrophic factor, NT‐3 and GDNF promote auditory neuron survival, axon extension and targeting to HCs.^24^, ^25^, ^26^ The spatiotemporal regulatory network of multiple TFs guides the SGN development. At E8.5, the sensory area of the auditory epithelium transiently upregulates the expression of Neurog1 followed by Neurod1 and differentiates into neurons. Neurog1 induces otic neurogenesis, while Atoh1 promotes differentiation into HC fate.^27^ Moreover, Eya1 or Six1 knockout initiates neurogenesis in early development, but is not able to form the cochlear vestibular ganglia.^28^, ^29^, ^30^ In addition, recent studies showed that Eya1 and Six1 complex activates Neurog1 and Neurod1.^31^ Hence, Neurog1 and Atoh1 have antagonistic effects on SGNs and HC fate conversion, and Eya1 and Six1 jointly regulate differentiation and maintenance of SGNs fate during the development of the inner ear. Pou4f1 is expressed at the stage of E9.5 sensory epithelial precursor cells (prosensory epithelia). After birth, Pou4f1 is gradually limited to the expression of partial SGNs and regulates presynaptic HC calcium ion signal activation.^32^ The expression of Pou4f2 starts after Pou4f1 and is maintained in SGNs.^33^ Islet1 is expressed at the E10.5 sensory epithelial precursor cell stage, and then expressed and maintained in auditory neurons^33^, ^34^ and involved in the specialization of the nervous system.^35^ In addition, previous studies showed the maturity and axon targeting of SGN are abnormal in Gata3 knocked‐out mice^36^ and restoration of Mafb rescues the synapse defect in Gata3 mutants.^37^ Therefore, understanding and manipulation of the developmental signals and TFs may pave a way to induce SGN regeneration.

TFs including Ascl1 and Neurod1 had been used to reprogramme sensory epithelium and glial cells to neurons in vitro.^38^, ^39^ Recently, two groups revealed that Neurog1/Neurod1 or Lin28 reprogrammed Plp1^+^ glial cells into cochlear neurons in neonatal mice in vivo.^40^, ^41^ These Neurog1/Neurod1 iNs showed the expression of neuron markers and SGNs markers, and decreased the expression of glial cell marker.^41^ Lin28 overexpression induced cochlear glial cells to express neuronal stem cell markers in SGN‐damaged mouse model and subsequent converted glial cells into neurons.^40^ However, these iNs show general neuronal characteristics while lacking SGN identity and function. In addition, both Neurog1/Neurod1 or Lin28 reprogramming efficiency is limited and significantly declined with ageing. As previous studies show that Neurog1 to Neurod1, Gata3 to Mafb may also have combined effects for early SGN development.^37^ The TF regulatory network for functional SGN reprogramming awaits further investigation and systematic screening of TF combinations may be a viable approach for SGN reprogramming.

Here, we applied systematic TF screenings and identified specific TFs combination Ascl1/Pou4f3/myt1l (APM) to induce multiple SGN marker expressions (neuronal, synaptic, ion channels) from mouse embryonic fibroblasts (MEFs). APM‐iNs exhibited more mature electrophysiological properties that are similar with SGNs. Thus, the overexpression of APM could serve as a promising strategy for directing fate conversion towards functional SGNs.

## 2 RESULTS

### 2.1 Ascl1 promotes Forskolin, ISX9, I‐BET151 and CHIR99021‐induced SGN fate conversion from MEFs

MEFs, as somatic cells with high ability of proliferation, are widely distributed and easy to obtain in large throughput. Combination of small molecule Forskolin, ISX9, I‐BET151 and CHIR99021 (FIBC) induced neuronal differentiation from MEFs efficiently.^42^, ^43^ Thus, MEF serves as an excellent cell model for high‐throughput screening of TFs for neuronal reprogramming.

We first performed concentration gradient screening of the small molecules of FIBC to identify the optimal concentrations of FIBC for neuronal differentiation from Tubb3‐mCherry MEF cells (Figure S1A). We examined the effects of small molecules on bipolar neuronal morphology, percentage of Tuj1^+^ cells and minimal cytotoxicity, and identified the optimal concentrations of Forskolin (20 μM), ISX9 (20 μM), I‐BET151 (1 μM) and CHIR99021 (10 μM) (Figure S1B).

**FIGURE 1:**
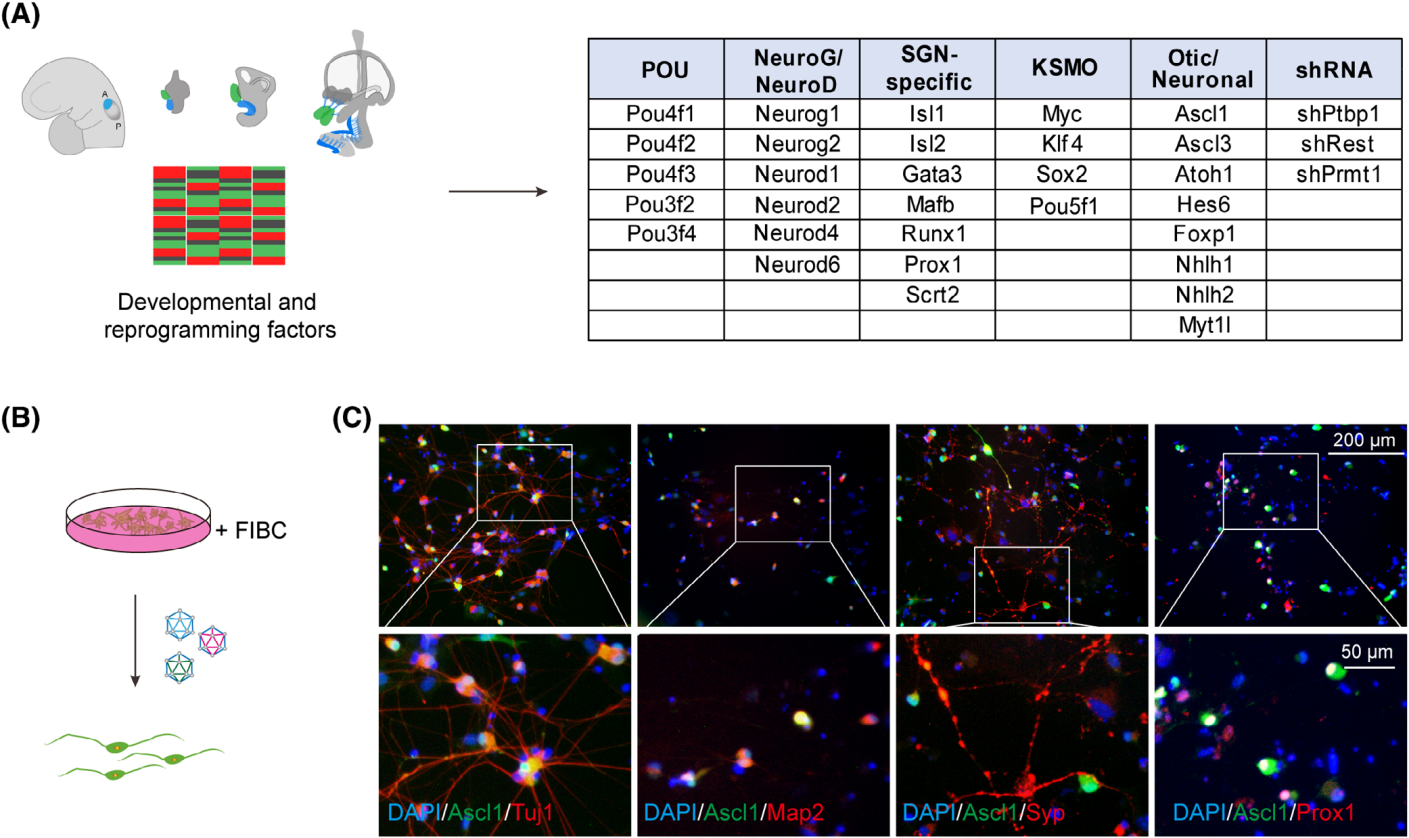
Ascl1 is a key pioneer transcription factor (TF) promoting Forskolin, ISX9, I‐BET151 and CHIR99021 (FIBC)‐induced reprogramming to spiral ganglion neuron (SGN)‐like neurons. (A) List of 33 potential reprogramming regulators, including 30 TFs and three negative regulators selected based on the SGN development and neuronal reprogramming studies. The 30 TFs were overexpressed and the three negative regulators were knocked down using lentivirus carrying ORFs or shRNAs, respectively. (B) Experimental design for MEFs co‐treated of FIBC and individual lentivirus modulating each reprogramming regulator. (C) Immunofluorescent images of Tuj1, Map2, Syp and Prox1 expression in induced neurons reprogrammed by FIBC and Ascl1. Lower panels were enlarged the images of the inserts from upper panels.

Based on the RNA sequencing analysis and previously reported omics data on SGNs,^14^, ^15^, ^16^, ^17^, ^44^, ^45^, ^46^ we shortlisted 33 TFs or neuronal repressors that may potentially regulate SGN fate (Figure 1A). The TFs or neuronal repressors were either overexpressed or knocked down with shRNA using lentiviral vectors in MEFs. We first induced the neuronal differentiation of MEFs with either of the 11 neuronal TFs (Ascl1, Mafb, Gata3, Pou3f4, Prox1, Hes6, Neurog2, Neurod1, Pou4f1, Pou4f2 and Runx1) in the presence of FIBC (Figure 1B). The results showed that Ascl1 was the sole TF with profound effect on increased cell survival and neuronal number. Furthermore, in the presence of FIBC, the Ascl1‐iNs expressed neuronal markers Tuj1 (Tubb3) and Map2, synapse marker Syp and SGN marker Prox1 (Figure 1C), while Syp and Prox1 expressions were not observed in iNs treated with FIBC alone. These results showed Ascl1 is a key pioneer TF, promoting FIBC‐induced neuronal survival, maturation and SGN fate conversion. Therefore, for the subsequent screening studies, we coupled Ascl1 with the remaining 32 TFs or neuronal repressors to examine potential synergistic effects on SGN reprogramming.

### 2.2 TF screening identifies Ascl1/Pou4f1 in promoting SGN reprogramming and maturation

While Ascl1 with FIBC can induce neuronal differentiation, neurons generated by Ascl1 alone rarely express mature or SGN‐specific markers, necessitating the identification of additional cooperating TFs. The inner ear's anatomy and blood–labyrinth barrier pose challenges for small molecule delivery and maintenance of therapeutic concentrations. TF‐based gene therapy offers advantages over small molecules, including direct cellular delivery, sustained potency and potentially reduced off‐target effects. Therefore, we conducted subsequent screening rounds without FIBC to develop a TF‐based reprogramming approach.

**FIGURE 2:**
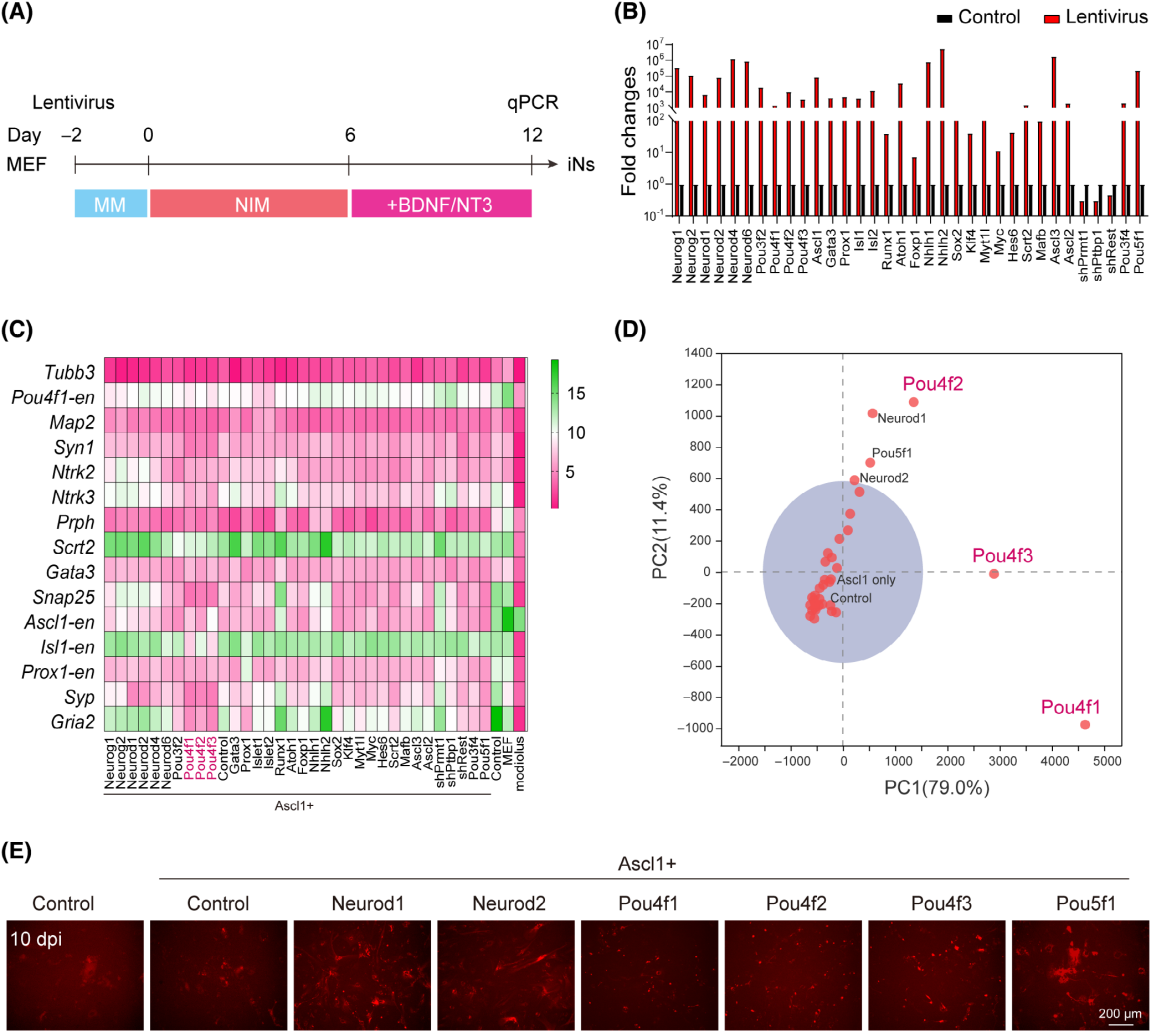
qPCR array and principal component analysis (PCA) identify Pou4f1, Pou4f2 and Pou4f3 to promote spiral ganglion neuron (SGN) fate conversion in combination with Ascl1. (A) Schematic illustration of the processes for neuronal reprogramming of Ascl1 + 1 induction. Mouse embryonic fibroblasts (MEFs) were infected with Ascl1 + each individual lentivirus for 2 days in MEF media (MM), followed by culture in neuronal induction media (NIM) for 6 days and in NIM with brain‐derived neurotrophic factor (BDNF) and NT3 for 6 days until analysis with qPCR array. (B) RT‐qPCR gene expression analyses of lentiviral overexpression of transcription factors (TFs) or knockdown of negative regulators. (C) Heat map of qPCR array for selected neuronal and SGN‐specific gene expression in induced neurons (iNs) by Ascl1 + 1 lentivirus at 12 days postinduction (dpi). (D) PCA of qPCR array data from Ascl1 + each of the 32 TFs or negative regulators. (E) Representative images the Tubb3‐mCherry iNs reprogrammed by the candidate induction conditions at 10 dpi.

To screening the effects of the remaining 32 TFs or repressors (Figure 1A), we performed qPCR array analysis of iNs at 12 days postinduction (dpi) (Figure 2A). The TF overexpression or repressor knockdown was confirmed by qPCR (Figure 2B). The gene expression array for evaluating SGN reprogramming includes pan‐neuronal markers and receptors (*Tubb3*, *Map2*, *Ascl1*, *Ntrk2* and *Ntrk3*), synaptic markers and receptors (*Syn1*, *Snap25*, *Syp* and *Gria2*) and SGN lineage markers (*Pou4f1*, *Prph*, *Scrt2*, *Gata3*, *Isl1* and *Prox1*). Endogenous expressions of *Pou4f1*, *Ascl1*, *Isl1* and *Prox1* were distinguished from the exogenous ones by targeting the UTR regions of these genes (Figure 2C). Principal component analysis (PCA) was performed on the gene expression array data to visualize the overall effects of each TF or repressor on SGN fate conversion (Figure 2D). PCA reduced the dimensions of these multiple gene expression data and increased interpretability with minimal information loss,^47^, ^48^ which was achieved by dividing the iNs from different TFs into parts, and transferring the difference of iNs into corresponding gaps from primary SGNs. POU4 family TFs (Pou4f1, Pou4f2 and Pou4f3), Neurod1, Neurod2 and Pou5f1 significantly altered the expression pattern of SGN marker genes (Figure 2D). In addition, neuronal differentiation efficiency of selected Ascl1 + 1 TF pairs was increased compared to that of Ascl1‐iNs (Figure 2E). Within the POU4 family, as the expression of Pou4f1 start from early otic development and maintained in the mature SGNs in adult, we selected Pou4f1 for further validations.

### 2.3 Ascl1/Pou4f1 promotes neuronal reprogramming towards SGN fate

**FIGURE 3:**
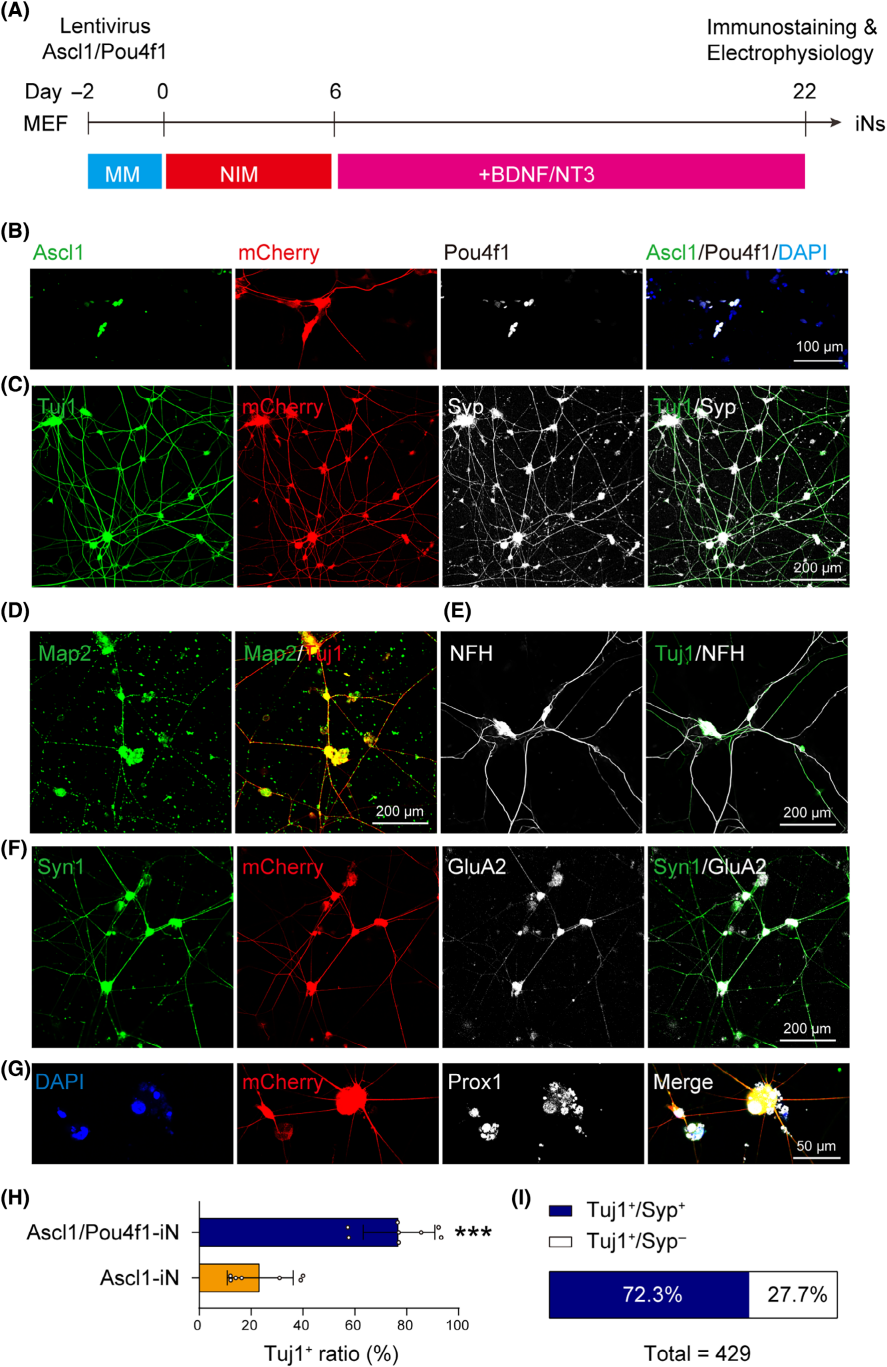
Induced neurons by Ascl1/Pou4f1 (AP‐iNs) exhibit molecular hallmarks of the spiral ganglion neuron lineage. (A) Schematic illustration of the neuronal induction process. (B) Immunofluorescent images of Ascl1 and Pou4f1 from iNs co‐infected with Ascl1/Pou4f1. (C–G) Immunofluorescent images of (C) Tuj1 and Syp, (D) Tuj1 and Map2, (E) Tuj1 and NFH, (F) Syn1 and GluA2, and (G) Prox1 with Tubb3‐mCherry from iNs induced with Ascl1/Pou4f1 at 22 days postinduction. (H) Ratio of Tuj1^+^ neurons induced by Ascl1 alone or Ascl1/Pou4f1. *N* = 7–8 random fields from three coverslips. Error bars represent mean ± SD. ***p < 0.001 by unpaired student's *t*‐test. (I) Ratio of Tuj1 single‐positive and Tuj1/Syp double‐positive neurons induced by Ascl1/Pou4f1. *N* = 7–8 random fields from three coverslips.

To detect the protein expression of the markers related to neuronal differentiation and maturation in iNs by Ascl1/Pou4f1 (AP‐iNs), immunocytochemical stainings were performed (Figure 3A). Ascl1, Pou4f1 and mCherry were colocalized in the iNs, confirming the proper overexpression of both Ascl1/Pou4f1 after lentiviral infection (Figure 3B).

The iNs gradually exhibited neuronal morphologies and expressed neuronal marker Tuj1 from 6 dpi and showed axonal elongation and neuronal maturation with longer induction time. We observed Tubb3‐mCherry, Map2 and NFH‐positive AP‐iNs with neuronal axon morphologies at 20 dpi (Figure 3C–E). In addition, the Tubb3‐expressing AP‐iNs were co‐labelled with mature neuron markers Syp and Syn1, suggestive of potential synaptic maturation in vitro (Figure 3C,F). Furthermore, AP‐iNs expressed the neuronal marker GluA2, suggesting that the AP‐iNs were glutamatergic (Figure 3F). Importantly, the AP‐iNs also expressed the SGN marker Prox1, indicative of induction towards SGN fate (Figure 3G). Then, we counted the ratio of Tubb3‐mCherry‐positive iNs with neuronal axon morphologies (Figure 3H). Percentage of Tuj1‐positive neurons were significantly more in AP‐iNs (76%) than those of Ascl1‐iNs (23%). Furthermore, the Tuj1/Syp double‐positive neurons accounted for 72.3% of AP‐iNs (Figure 3I). These findings suggest that Ascl1/Pou4f1 is a key TF pair to promote neuronal differentiation, maturation and SGN fate conversion.

**FIGURE 4:**
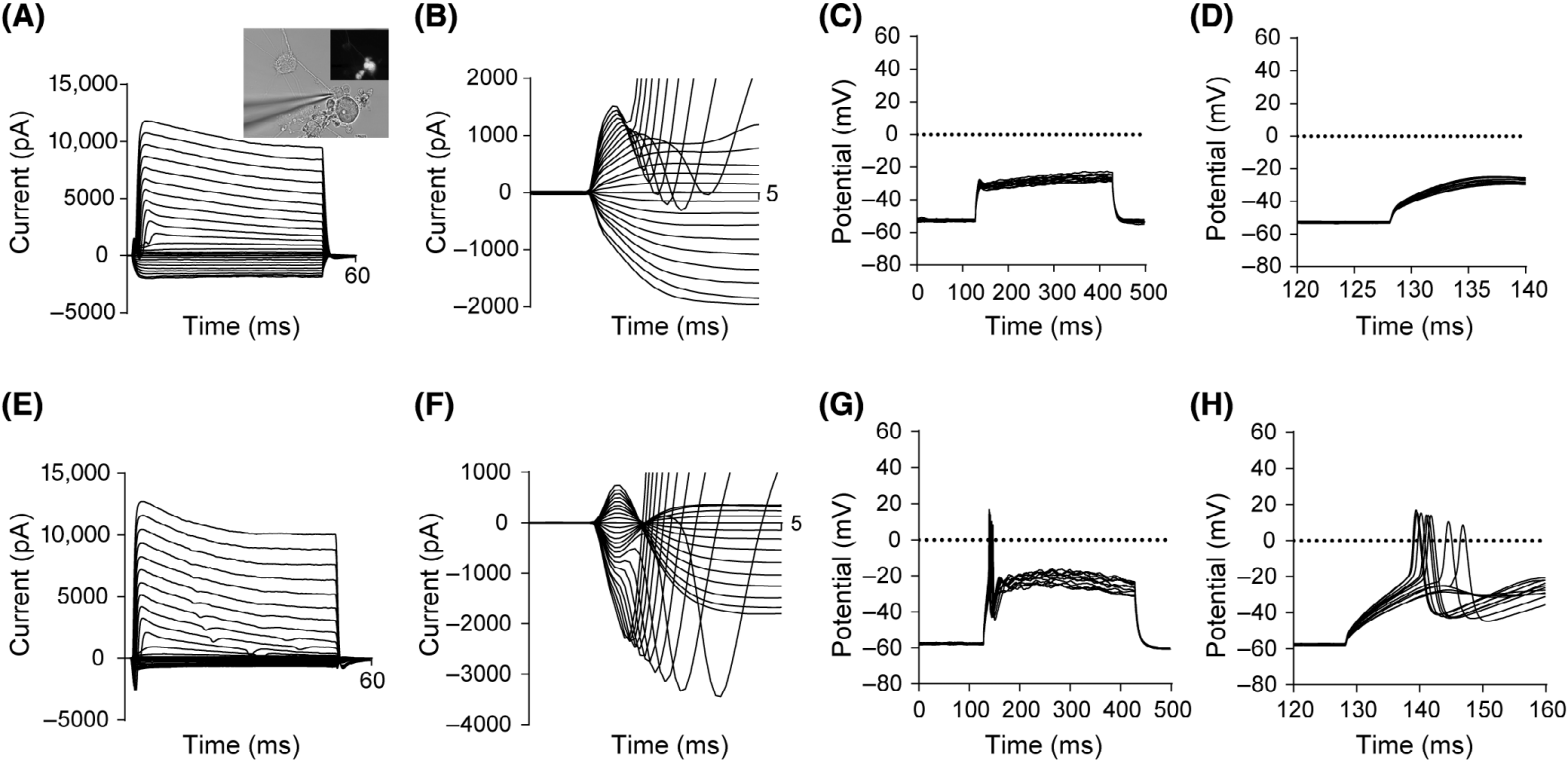
Induced neurons (iNs) by Ascl1/Pou4f1 exhibit functional properties of sensory neurons. (A, B) Voltage‐clamp recordings of (A) K^+^ currents and (B) Na^+^ currents at 12 days postinduction (dpi). Micrograph showed a typical iNs chosen for patch‐clamp recordings. (C, D) Representative traces showing action potentials of iNs at 12 dpi without electrophysiological properties. (E, F) Voltage‐clamp recordings of (E) K^+^ currents and (F) Na^+^ currents at 22 dpi. (G, H) Representative traces showing action potentials of iNs at 22 dpi with naive single‐spiking electrophysiological properties.

Next, the whole‐cell patch‐clamp recordings were performed to examine the electrophysiological properties of the AP‐iNs with bipolar neuronal morphology. We did not detect the potassium (K^+^) or sodium (Na^+^) currents in MEFs infected with control lentivirus. Interestingly, AP‐iNs generated K^+^ currents and small Na^+^ currents but no APs, suggesting that they were functionally immature at 12 dpi (Figure 4A–D). At 22 dpi, majority of neurons (three of four) had typical Na^+^ and K^+^ currents, which corresponded to the opening of voltage‐dependent sodium and potassium channels (Figure 4E,F). Remarkably, one of the recorded AP‐iNs exhibited AP responses (Figure 4G,H). Thus, the AP‐iNs appeared to exhibit the naive functional membrane properties and electrophysiological properties of neurons.

### 2.4 Ascl1/Pou4f1/Myt1l promotes SGN differentiation and maturation

**FIGURE 5:**
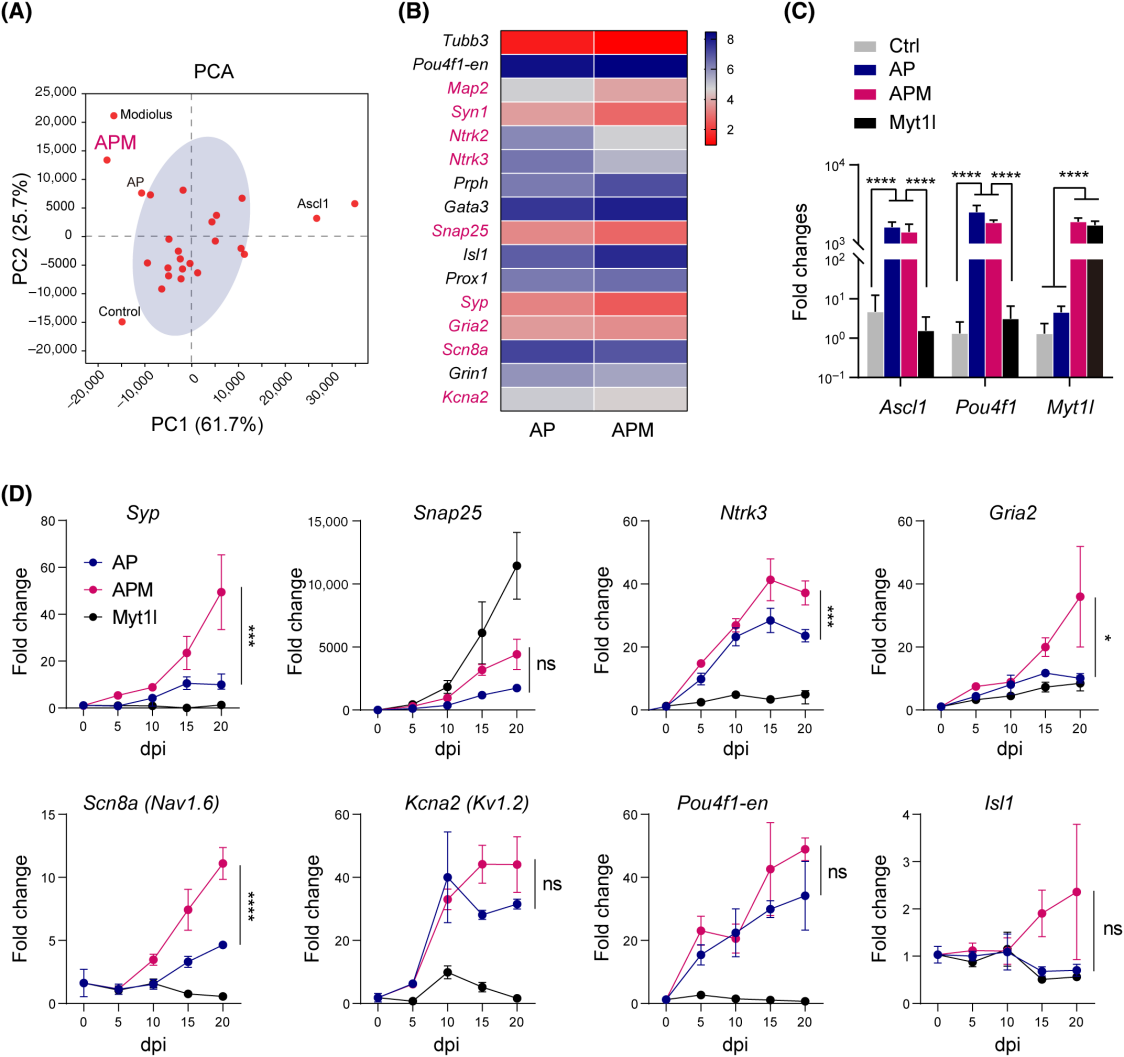
Myt1l further promotes expression of spiral ganglion neuron (SGN) markers in combination with Ascl1/Pou4f1. (A) Principal component analysis (PCA) of qPCR array data from Ascl1/Pou4f1 + each of the 31 transcription factors or negative regulators. (B) Heat map of qPCR array for selected neuronal and SGN‐specific gene expression in neurons induced by Ascl1/Pou4f1 (AP) or Ascl1/Pou4f1/Myt1l (APM) at 12 days postinduction (dpi). Differentially regulated genes were highlighted in magenta. (C) RT‐qPCR gene expression analyses of lentiviral overexpression of Ascl1, Pou4f1 and Myt1l in iNs at 12 dpi. ****p < 0.0001 by one‐way ANOVA. (D) Temporal induction of neuronal and SGN‐specific genes in neurons induced by AP, APM or Myt1l alone. *p < 0.05, ***p < 0.001 and ****p < 0.0001 by two‐way ANOVA. ns, not significant.

The naïve electrophysiological characteristics of AP‐iNs suggest other TFs may be required for further maturation of the iNs towards functional SGNs. We then screened the effects of the remaining 31 TFs and repressors in the presence of Ascl1 and Pou4f1. Gene expression array and PCAs showed that Myt1l enhanced neuron conversion in the presence of Ascl1/Pou4f1 (APM), compared to AP‐iNs (Figure 5A,B). Myt1l expression was assessed in the mouse cochlea, and RT‐qPCR and immunofluorescence staining revealed its specific expression in SGN, which increased with age (Figure S2). To assess the temporal effects of Myt1l on neuronal differentiation towards SGN lineage, we examine the dynamic expression of neuronal markers at various time points after overexpression of AP, APM or Myt1l alone. Overexpression of APM was validated by qPCR analysis (Figure 5C). APM‐iNs showed significant upregulation of *Syp*, *Ntrk3*, *Gria2* and *Nav1.6* expressions compared to AP‐iNs (Figure 5D). The results demonstrated that compared to AP, co‐induction of APM enhanced the expression of mature SGN markers.

**FIGURE 6:**
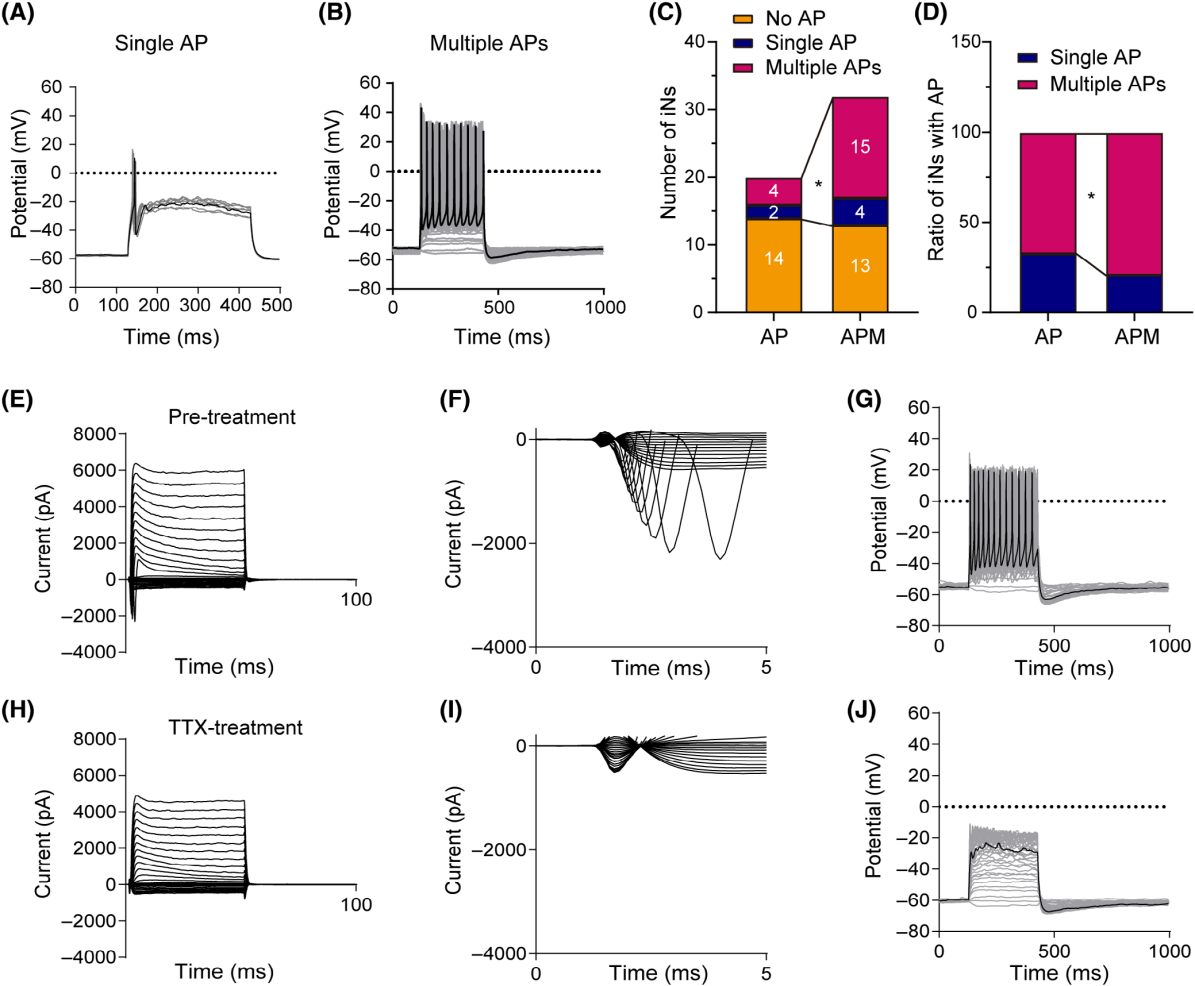
Ascl1/Pou4f1/Myt1l (APM)‐induced neurons exhibit more mature electrophysiological properties. (A, B) Representative electrophysiological traces showing (A) single or (B) multiple action potentials (APs). (C, D) APM induced more neurons (C) firing action potentials and (D) firing multiple APs. *p < 0.05 by Chi‐square test. (E–J) Voltage‐clamp recordings of (E, H) K^+^ currents, (F, I) Na^+^ currents and (G, H) APs in APM‐induced neurons (E–G) before and (H–J) after tetrodotoxin (TTX) treatment.

The whole‐cell patch‐clamp recordings showed that APM‐iNs displayed both single and multiple APs (Figure 6A,B). Remarkably, compared to AP‐iNs, a greater number of APM‐iNs generated APs (Figure 6C) and specifically multiple APs (Figure 6D). Tetrodotoxin is a highly selective, rapidly reversible sodium channel blocker that prevents the influx of sodium ions into the cell, thereby influencing the cell membrane AP. Our findings indicated that tetrodotoxin treatment could suppress the electrophysiological characteristics of APM‐iNs (Figure 6E–J), suggesting the presence of typical Na^+^ channels.

**FIGURE 7:**
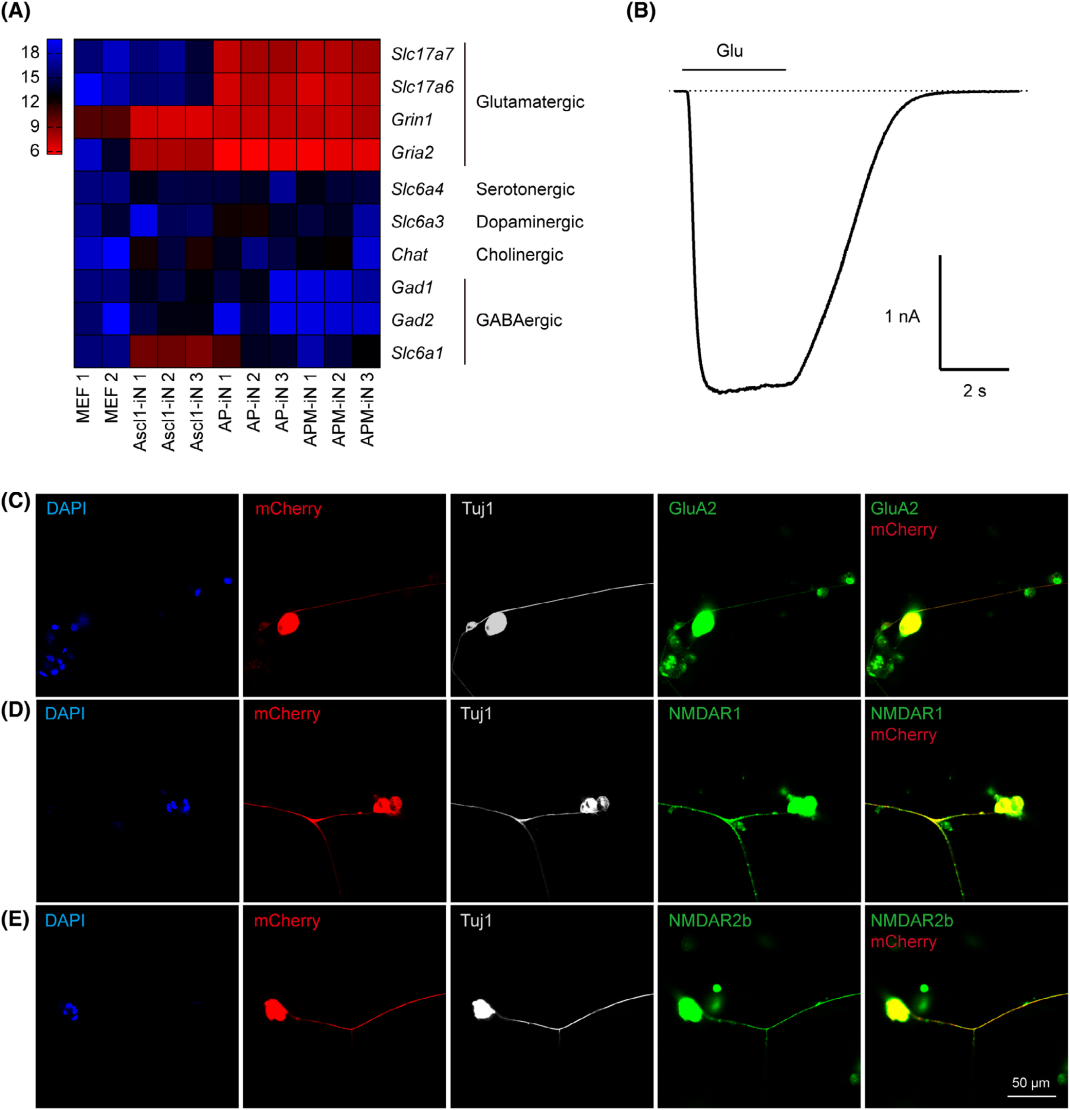
Ascl1/Pou4f1/Myt1l‐induced neurons (APM‐iNs) are glutamatergic and respond to glutamate. (A) Heat map of qPCR data showing expression of glutamatergic, serotonergic, dopaminergic, cholinergic and GABAergic markers in mouse embryonic fibroblasts (MEFs) and neurons induced by Ascl1 alone, Ascl1/Pou4f1 (AP) or APM. (B) Electrophysiological recording of APM‐iNs responding to exogenous glutamate. (C–E) Immunofluorescent images of APM‐iNs‐expressing glutamatergic protein markers (C) GluA2, (D) NMDAR1 and (E) NMDAR2b.

To investigate the effects of the TFs on neuronal subtype specifications, we performed qPCR analysis of the subtype‐specific markers. We found that while Ascl1 alone promoted the expression of both glutamatergic and GABAergic markers, AP and APM induced expression of more glutamatergic markers and suppressed the expression of GABAergic markers (Figure 7A). Electrophysiological analysis also demonstrated the responsiveness of APM‐iNs to glutamate stimulation (Figure 7B). Immunofluorescence staining revealed the expression of various glutamatergic marker proteins in APM‐iNs (Figure 7C–E). Consequently, APM‐iNs exhibit enhanced maturity in electrophysiological traits characteristic of auditory neurons, indicating the pivotal role of APM in promoting neuronal differentiation, maturation and the transformation of SGN fate through a key combination of TFs.

### 2.5 Ascl1/Pou4f1/Myt1l iNs exhibit transcriptomic profiles similar to the endogenous SGNs

**FIGURE 8:**
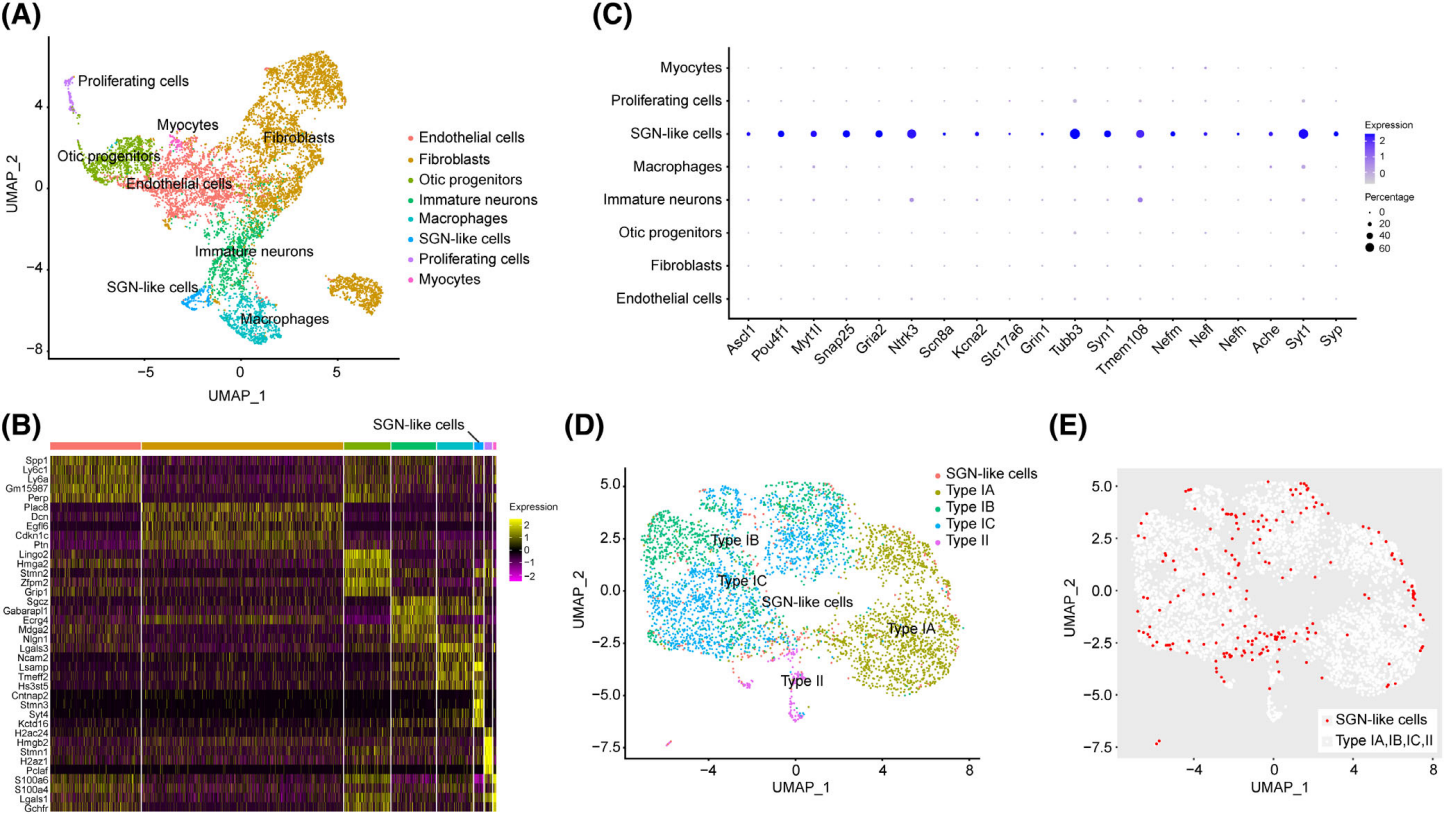
Ascl1/Pou4f1/Myt1l‐induced neurons (APM‐iNs) display single‐cell transcriptomes similar to the endogenous spiral ganglion neurons (SGNs). (A) UMAP plot of RNA samples obtained from single cells of APM‐iNs at 20 days postinduction. Eight different clusters were identified. (B) Heat map showing the top five differentially expressed genes among clusters. (C) Genes specifically expressed by SGN‐like cells shown in dot plots. (D) UMAP plot showing SGN‐like cells with adult SGNs.^15^ (E) SGN‐like cells evenly dispersed among SGN subtypes.

Single‐cell RNA sequencing (scRNA‐seq) was performed on iNs with co‐overexpression of APM at 20 dpi. Eight distinct clusters can be identified (Figure 8A,B), including two populations, both with the expression of neuronal genes. In one of the neuronal populations, adult SGN markers were selectively expressed (Figure 8C), and therefore, we named this cluster ‘SGN‐like cells’ and the other one ‘immature neurons’. Notably, cells with high level of APM expression almost exclusively differentiated into ‘SGN‐like cells’ (Figure 8C), suggesting co‐expression of the three TFs efficiently induces SGN differentiation. To examine whether the SGN‐like cells resemble endogenous SGNs, we compared their transcriptome with adult SGNs.^15^ SGN‐like cells did not clearly adopt a Type Ic fate (Figure 8D,E), despite overexpression of Type Ic specific TF Pou4f1 (Figure 8C). Instead, they were intermingled with multiple SGN subtypes on UMAP plot (Figure 8D,E), confirming their transcriptomic similarity to endogenous SGNs.

**FIGURE 9:**
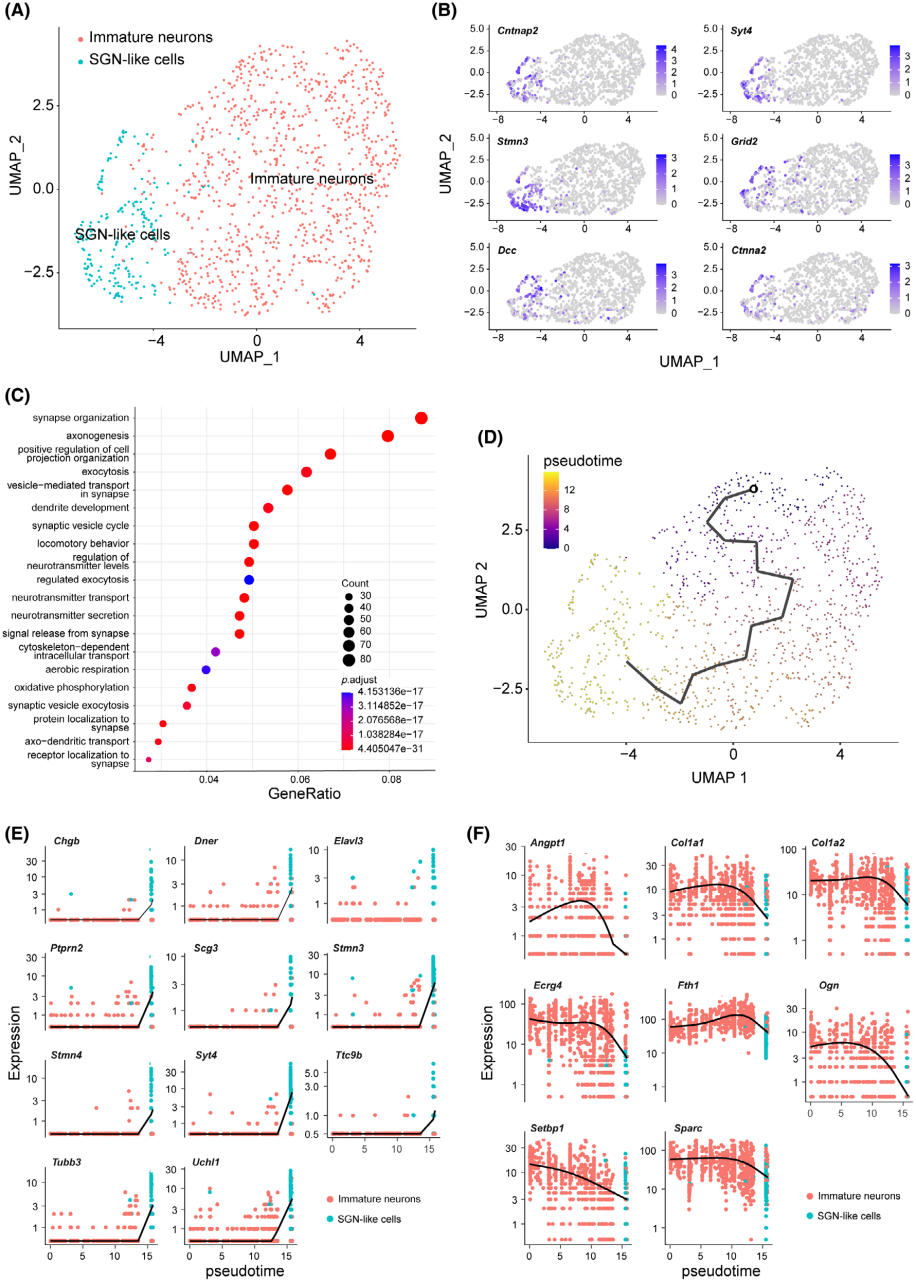
Spiral ganglion neuron (SGN)‐like cells from Ascl1/Pou4f1/Myt1l‐induced neurons may be derived from transitional immature neurons. (A) UMAP plots with SGN‐like cells and immature neurons. (B) Feature plots showing genes enriched in SGN‐like cells compared with immature neurons. (C) GO pathway enrichment analysis of differentially expressed genes upregulated in SGN‐like cells compared with immature neurons. (D) A pseudotime analysis with Monocle. A trajectory detected with root in immature neurons and outcome in SGN‐like cells. (E, F) Pseudotime kinetics of genes (E) upregulated and (F) downregulated in SGN‐like cells, respectively.

In addition to expression of SGN‐specific markers, the ‘SGN‐like cells’ also express more mature neuronal genes (Cntnap2, Syt4, etc.) than the ‘immature neurons’ (Figure 9A,B). Consistently, GO pathway enrichment analysis showed that compared to the ‘immature neurons’, the ‘SGN‐like cells’ upregulated genes related to synaptic organization, axonogenesis, dendritic development, etc. (Figure 9C), suggesting that the ‘SGN‐like cells’ may be derived from the ‘immature neurons’. Indeed, pseudotime analysis with Monocle3 identified a lineage starting from ‘immature neurons’ and terminates in ‘SGN‐like cells’ (Figure 9D–F). Consistently, RNA velocity (Figure S3A,B **)** and CytoTRACE (Figure S3C,D) analyses also yielded similar results. These results suggest that immature neurons may represent a transitional state during SGN reprogramming, which is promoted by APM expression.

## 3 DISCUSSION

**FIGURE 10:**
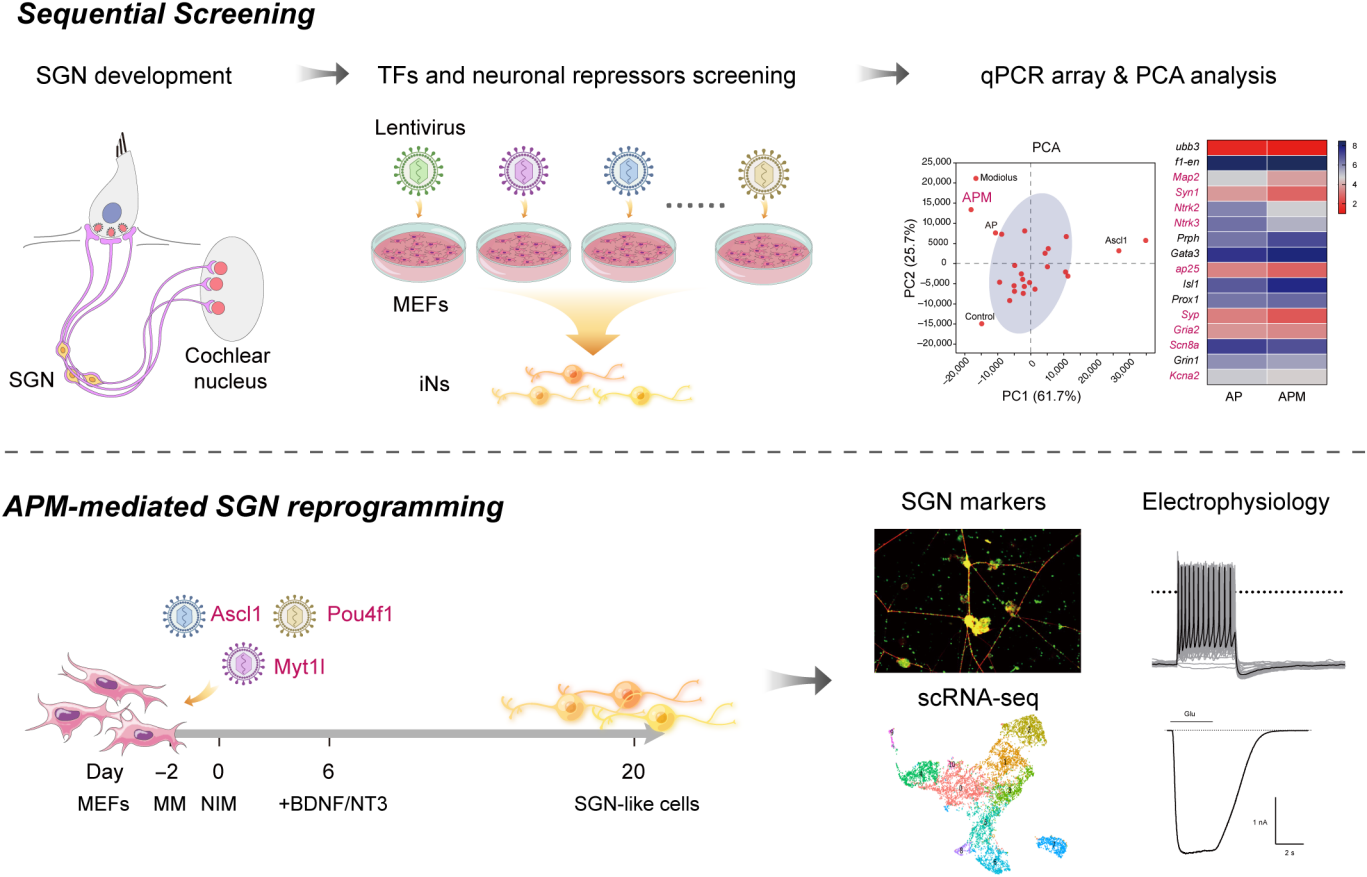
Graphic illustration of spiral ganglion neuron (SGN) reprogramming by Ascl1/Pou4f1/Myt1l (APM). An unbiased sequential screening of the 33 neuronal regulators was performed on mouse embryonic fibroblasts (MEFs) and reprogramming towards SGN fate was evaluated by qPCR array followed by principal component analysis (PCA). A combination of APM was identified to promote functional reprogramming of SGNs based on biochemical, electrophysiological and single‐cell transcriptomic analyses. AP, Ascl1/Pou4f1; BDNF, brain‐derived neurotrophic factor; iNS, induced neurons; MM, MEF media; NIM, neuronal induction media; scRNA, single‐cell RNA sequencing; TF, transcription factor.

In this study, we screened 33 potential TFs and neuronal repressors to identify novel factors in promoting SGN reprogramming and maturation. We also developed an approach with SGN fate‐specific qPCR array followed by PCA as a quantitative readout to globally evaluate the neuronal maturation and SGN identity of the iNs. Through sequential screening, we identified a specific combination of TFs consisting of APM that induce reprogramming of MEFs into SGN‐like cells. These APM‐iNs express mature neuronal and SGN‐specific markers, display SGN‐like electrophysiological properties and exhibit similar transcriptomic signatures as the endogenous SGNs (Figure 10).

TFs are widely used for cell lineage reprogramming, such as iPSCs (Sox2, Klf4, c‐Myc and Oct4),^49^, ^50^ monocytes (PU.1 and C/EBPβ),^51^, ^52^ cardiomyocytes (GATA4, Mef2c and Tbx5),^53^ hepatocytes (Hnf4α, Foxa1, Foxa2 and Foxa3),^54^ myoblasts (MyoD)^55^ and neuronal progenitors (Oct4, Sox2, Klf4 and c‐Myc).^56^ Pioneer TFs and lineage‐specific TFs have also been shown to reprogram fibroblasts or glia cells into different neuronal subtypes,^11^ including dopaminergic,^57^, ^58^ motor,^59^, ^60^ GABAergic,^61^ sensory^62^ and retinal ganglion neurons.^63^

Studies on cochlear SGN reprogramming are currently limited. Recently, Ascl1/Neurod1 and Neurog1/Neurod1 were used in cochlear neuron reprogramming, while these iNs showed general neuronal characteristics but lacked SGN identity and function.^40^, ^41^ Based on our finding, it is likely that Pou4f1, an early otic neuron lineage TF that later restricted in type 1c SGNs,^32^ may be essential to establish SGN fate during reprogramming. Interestingly, conditional deletion of *Pou4f1* in SGNs did not alter the developmental patterning of SGN or synaptic innervations, but only influenced the voltage dependence and intensity of presynaptic Ca^2+^ influx.^32^ Therefore, while Pou4f1 is required for SGN differentiation earlier on, whether it instructs the subtype specification of type 1c SGNs remains elusive.

Furthermore, the finding that Myt1l further promotes mature neuronal marker expression and multiple AP firing suggests that Myt1l is required for functional maturation of the lineage‐converted SGN‐like cells. Human genetic research has demonstrated that mutations leading to the loss of function in Myt1l are associated with neurodevelopmental disorders.^64^ Myt1l mRNA levels rise throughout neurogenesis in mice, maintaining lower levels into adulthood, reflecting expression patterns observed in humans.^65^ Numerous studies indicate that Myt1l, along with other members of the MYT family, primarily serves to maintain neuronal phenotypes. This role is supported by findings showing that Myt1l is largely expressed during the postspecification phase when cell populations have transitioned into a postmitotic state.^64^ Our result is consistent with an important role of Myt1l in safeguarding neuronal identity and promoting neuronal maturation during reprogramming.^66^, ^67^

Multiple studies of neuron regeneration by in vivo reprogramming have been achieved by ectopic expression of lineage‐determining TFs^68^, ^69^, ^70^, ^71^, ^72^, ^73^, ^74^ or through knockdown of neuronal inhibiting gene,^75^, ^76^ some of which also demonstrated certain therapeutic values in damaged or pathological conditions.^68^, ^72^, ^74^, ^75^, ^76^, ^77^ Recent advances in inner ear gene therapy have yielded promising clinical results, demonstrating that adeno‐associated virus‐mediated gene replacement therapy is safe and can restore hearing in patients with profound hereditary hearing loss.^78^, ^79^, ^80^, ^81^ Gene delivery by adeno‐associated virus has also been shown to promote regeneration and protection of HCs and SGNs in mouse models.^82^, ^83^ In addition to the genetic modulations, recent studies also highlight the effectiveness of tissue engineering approaches to support growth and function of regenerated SGNs.^84^, ^85^, ^86^ The cochlear resident non‐neuronal cells (glia or fibroblasts) can replenish themselves and provide initial cell sources for reprogramming by proliferation followed by differentiation, including at sites of SGN injury or degeneration. Therefore, based on the combination of reprogramming TFs screened in our in vitro system, combined with tissue engineering strategies and genetic approaches to regulate gene expression in vivo, it may be possible to reprogramme cochlear resident glial cells or fibroblasts into functional SGNs.

Transcriptomic and SGN electrophysiological assessments are reliable readouts for evaluation and interrogation of SGN reprogramming for functional recovery of newly generated iNs in vitro.^11^ However, functional neuronal regeneration not only requires the iNs to replace the damaged or lost neurons, but also requires re‐establishment of neuronal circuit with functional synapses between the iNs and HCs or cochlear nucleus neurons, and also proper myelination of the iN axons.^8^ Currently, majority of the in vivo studies did not test circuit integration and recovery of auditory functions. Future studies with in vivo directly reprogramming of SGNs should also face and overcome the challenge of functional synaptic re‐connections between SGNs and HCs, as well as SGNs and cochlear nucleus.

## 4 MATERIALS AND METHODS

### 4.1 Animals

Tubb3‐mCherry mice were generated as previously reported.^87^ All mice used in this work were on a mixed background containing C57BL6 and FVB/N strains. Both male and female mice were used. All animal procedures were approved by the Institutional Animal Care and Use Committee of Model Animal Research Center of Nanjing University.

### 4.2 Plasmid constructions

Mouse cDNA fragments of the 30 TFs were cloned into lentiviral constructs of pLKO.1‐puro vector (#8453, Addgene). Lentiviral shRNAs against mouse *Rest*, *Ptbp1* and *Prmt1* were cloned in the pLKO.1 vector. The shRNA target sequences were as follows: shPrmt1, 5′‐GCTGAGGACATGACATCCAAA‐3′; shRest, 5′‐GTGTAATCTACAATACCATTT‐3′; shPtbp1, 5′‐GCGGGTGAAGATCCTGTTCAA‐3′. All mammalian expression plasmids were amplified in DH5α bacteria. All constructs were verified by sequencing and transfection.

### 4.3 Cell culture, lentiviral production and infections

HEK293T cell was cultured in Dulbecco's modified Eagle medium (DMEM) (12,800,017, Gibco) supplemented with 10% foetal bovine serum (40130ES76, Yeasen), GlutaMAX (35050079, Gibco), non‐essential amino acid (NEAA) (11140050, Gibco), sodium pyruvate (11360070, Gibco) and P/S (E607011‐0100, Sangon Biotech).

The lentiviral particles were generated by co‐transfecting each pLKO.1 core plasmids with two additional lentiviral packaging helper plasmids, pSPAX2 (#12260, Addgene) package and pMD2.G (#12259, Addgene) envelope plasmids, with PEIMAX‐40K (24765, Polysciences) in HEK293T cells (Cell Resource Center, Peking Union Medical College). The culture supernatant was collected at 24–48 h after transfection and then passed through a 0.45‐μm strainer to remove the cell debris. Viruses were concentrated from culture supernatant by ultra‐centrifugation (25,000 rpm, 2 h, 4°C). After 24–48 h infection of cells with 10 μg/mL polybrene (sc‐134220, Santa Cruz Biotech), virus‐containing medium was replaced with fresh media.

### 4.4 Preparation of MEF

The MEFs were prepared as previously described.^88^ For isolation of MEFs, pregnant female mouse at E13.5 or E14.5 was anaesthetized and the embryos transferred to a 10‐cm culture dish containing cold Hanks' balanced salt solution (354232, Gibco). The brain and internal organs of each embryo were removed using a sterilizing razor in fresh 60‐mm petri dish with 5 mL Hanks' balanced salt solution on ice under a dissecting microscope.

The remaining tissue was transferred into a fresh 35‐mm culture plate containing 1 mL of 0.25% trypsin–ethylene diamine tetra‐acetic acid (59418C, Sigma), thoroughly minced using a pair of surgical scissors and forceps, and then incubated for 1–2 h in a 15‐mL conical tube at 37°C with shaking. After being mixed with 5 mL of MEF media (DMEM, 10% foetal bovine serum and 1% P/S) using a 10‐mL pipette, the digested tissue was transferred to a 15‐mL fresh tube, centrifuged at 1200 g for 5 min at room temperature and resuspended in 10‐mL fresh MEF medium at 37°C in a CO_2_ incubator.

### 4.5 Neuronal differentiation

The induction media for small molecule reprogramming contains Neurobasal Medium (21103049, Gibco), supplemented with B27 (17504044, Gibco) and N2 (17502048, Gibco), GlutaMax, P/S and bFGF (450–33, PeproTech), with or without small molecules Forskolin (S2449, Selleck), ISX9 (S7914, Selleck), I‐BET (S2780, Selleck) and Chir99021 (S1263, Selleck). Differentiated cells were analysed after 8 or 18 days for immunocytochemistry and qPCR.

For neuronal reprogramming induced by TFs, MEFs were plated on glass slides (12‐545‐80, Thermo Fisher) coated with 10 ng/mL Laminin (354232, Corning). After 24‐h infection, the virus and medium mixture was removed. The cells were induced for 4 days in the neuron induction media containing DMEM/F12 (12634010, Gibco), supplemented with B27 and N2, GlutaMax, NEAA, P/S and bFGF. Then, for following induction, the iNs were induced in the neuron maintenance medium containing of a 1:1 mix of DMEM/F12, Neurobasal Medium, supplemented with B27 and N2, GlutaMax, NEAA, penicillin–streptomycin and bFGF, brain‐derived neurotrophic factor (78005, Stemcell) and NT3 (78074, Stemcell). The iNs were analysed at the indicated time for qPCR, immunocytochemistry and electrophysiological recordings.

### 4.6 Real‐time quantitative PCR

The induced cells were homogenized and total RNA extracted by RNAiso Plus kit (9109, Takara). The quantity and purity of samples were detected by determining absorbance at 260/280 nm by Nanodrop. Reverse transcription of total RNA was performed with the PrimeScript RT reagent kit (RR047A, Takara) according to the manufacturer's protocol. The Quantitative PCRs were performed with the Hieff UNICON® qPCR SYBR Green Master Mix (11198ES03, Yeasen) on LightCycler® 96 Instrument (05815916001, Roche). Data are normalized to GAPDH, and fold changes are calculated by using 2^−ΔΔCT^ method. Details of the primers are listed in Table S1.

### 4.7 Immunofluorescence

Cells were fixed in 4% paraformaldehyde in phosphate‐buffered solution (PBS) for 15 min with shaking at room temperature. Isolated cochleae of mice were fixed in 4% paraformaldehyde in PBS for 2 h with shaking at room temperature, followed by decalcification in 5% ethylene diamine tetra‐acetic acid for 4–5 days. Cells or the dissected cochlea were blocked with 5% heat‐inactivated horse serum with 0.3% Triton X‐100 in PBS for 1 h.

iNs were incubated with primary antibody overnight at 4°C. The primary antibodies used in this study were as follows: mouse anti‐Tuj1 (1:2000, MMS 435P, Biolegend), goat anti‐Prox1 (1:250, AF2727, R&D), mouse anti‐Map2 (1:250, M4403, Sigma‐Aldrich), rabbit anti‐Syp (1:200, MA514532, Life Technologies), mouse anti‐Pou4f1 (1:200, MAB1585, Millipore), rabbit anti‐Ascl1 (1:500, 383536, Zenbio), rabbit anti‐Myt1l (1:200, 25234‐AP, Proteintech), rabbit anti‐Syn1 (1:500, 5297, Cell Signaling Technology), mouse anti‐GluA2 (1:2000, MAB397, Millipore), mouse anti‐Syp (1:300, MA5‐14532, Thermo Fisher), rabbit anti‐NMDAR2b (1:500, 21920‐1‐AP, Proteintech), rabbit anti‐NMDAR1 (1:500, ab109182, Abcam), chicken anti‐NFH (1:1000, AB5539, Millipore) and rabbit anti‐GFP (1:400, 31,002, Yeasen). Cells or tissues were then incubated with Alexa 488‐, Alexa 568‐ and/or Alexa 647‐labelled secondary antibodies (1:500, Jackson ImmunoResearch) for 1–2 h with shaking at room temperature. Nuclei were visualized with DAPI (1:2000, 28718‐90‐3, Roche).

Confocal z‐stacks (0.5 μm step size) of cochlear tissues were taken using a Leica SP5 confocal microscope (Leica TCS SP5, Leica Microsystems) or Zeiss LSM 800 confocal microscope (ZEN 2.1, Carl Zeiss) equipped with 40× and 63× oil immersion lens, and inverted fluorescence microscope (XD, SOPTOP). ImageJ software (version 1.52i, NIH, Bethesda, MD, United States) was used for image processing and three‐dimensional reconstruction of z‐stacks. All immunofluorescence images shown are representative of at least three individual results. Efficiency of conversion was measured by the number of Tuj1^+^ cells divided by the total number of plated cells from random 6–10 fields.

### 4.8 Single‐cell RNA sequencing

ScRNA‐seq on APM‐iNs was performed by Majorbio Bio‐Pharm Technology Co., Ltd. (Shanghai, China). All sequencing data are available through the NCBI Sequence Read Archive under the accession number PRJNA1136564. The raw reads of our transcriptome data have been deposited into the NCBI Short Read Archive under accession number SRR29849296. Sequence alignment was carried out using 10× Genomics CellRanger (v.7.0.0). The resulting feature–barcode matrix was filtered and visualized using Seurat (v.4.3.0). First, DropletUtils (v.1.18.0) and scDblFinder (v.1.12.0) were used for quality control, doublets and multiplets were screened out, and cells with a mitochondrial mapping greater than 6% were also removed. Next, the FindAllMarkers command was used to find the top markers that define the cell cluster's identity, based on which each cell cluster was named. The IntegrateData function was then used to integrate the published adult SGN dataset^15^ and the enriched SGN‐like cells cell cluster. In functional enrichment analysis, the enrichGO functions of clusterProfiler (v.4.6.2) are used to enrich GO enrichment analyses. In order to infer the degree of differentiation of different cell clusters in the dataset, CytoTRACE (v.0.3.3) was used to predict the degree of differentiation of different cell clusters. Next, the immature neuron and SGN‐like cell clusters are extracted from the data set, and Monocle3 (v.1.0.0) is used for trajectory inference and analysis. Based on the analysis results of CytoTRACE, the cell clusters with the lowest degree of differentiation are selected as nodes. The roots of the trajectory are specified and the trajectory is plotted. Then, Velocyto.R (v.0.6) was further used for RNA rate analysis to infer the fate state and differentiation direction of cell clusters, and the predicted differentiation trajectory was consistent with the differentiation direction of cells.

### 4.9 Electrophysiological analysis

For the electrophysiological recordings of iNs, MEFs for neuronal differentiation are mounted on glass coverslips and glass coverslips with adhered cells were transferred into a recording chamber. iNs were observed by upright microscope (Olympus, BX51W1, Japan) equipped with a 40× water‐immersion objective and differential interference contrast optics.

The voltage‐dependent currents and APs were recorded whole‐cell via current‐clamp mode and voltage‐clamp mode, respectively. In the recordings of the voltage‐dependent currents, the basal holding potential was −70 mV and −160 to +70 mV/50 ms stimulations with 10 mV stepping increment and 1‐s interval were used to evoke the currents. APs were induced by −10 to +400 pA/300 ms stimulations with 10 pA stepping increment and 5‐s interval. The recordings were performed at room temperature (22 ± 2°C) in the extracellular solution contained (mM): 145 NaCl, 5 KCl, 1 CaCl_2_, 1 MgCl_2_, 5 d‐glucose, 25 sucrose, 5 HEPES, pH 7.4 adjusted with NaOH and 300 mOsm/L osmolality. Recording electrodes (3–5 MΩ) were pulled using P1000 glass microelectrode puller (Sutter Instrument, USA) and filled with the internal solution contained (mM): 130 K‐gluconate, 10 KCl, 2 MgCl_2_, 0.5 EGTA, 2 Na_2_‐ATP, 0.3 Na_3_‐GTP, 10 HEPES, pH 7.4 adjusted with KOH, 290 mOsm/L osmolality. The electrophysiological data collected through a series of patch‐clamp instruments from Molecular Devices (USA), including a MultiClamp 700B amplifier, a Digidata 1550 digitizer and the correlated pClamp 10 software. The data were digitized at 10 kHz and filtered at 2 Hz.

### 4.10 Statistical analysis

Statistical tests were performed using GraphPad Prism 8 (GraphPad Software Inc., La Jolla, CA, United States). Results were reported as mean ± SD. Specific statistical tests used in each experiment were described in figure legends. Results were analysed using Student's *t*‐test or one‐way ANOVA, followed by Bonferroni's multiple comparisons test.

## AUTHOR CONTRIBUTIONS

ZC, XM, SS, YSS and GW conceived and designed the experiments. YH, ZC and JC performed most of the experiments. JL and SS performed the scRNA‐seq analyses. CQ, QL, LZ and G‐JZ assisted with the experiments. YH, ZC, XM, SS and GW wrote the manuscript with help from other authors.

## CONFLICT OF INTEREST STATEMENT

The authors declare no conflicts of interest.

## Supporting information


**Data S1.** Supporting information.

## Data Availability

Single‐cell RNA sequencing data are available through the NCBI Sequence Read Archive under the accession number PRJNA1136564. The raw reads of our transcriptome data have been deposited into the NCBI Short Read Archive nder accession number SRR29849296.
