# Supplementary material for: Direct reprogramming of fibroblasts into spiral ganglion neurons by defined transcription factors
Source: Cell Prolif. 2024 Nov 17;58(4):e13775. doi: 10.1111/cpr.13775 (PMC11969255; doi:10.1111/cpr.13775)
Supplement: Supplementary file 1 — Data S1. Supporting information. [file CPR-58-e13775-s001.docx]

**SUPPLEMENTARY MATERIALS**

**Direct reprogramming of fibroblasts into spiral ganglion neurons by defined transcription factors**

Huang et al.

**This file contains:**

Supplemental Figures S1-3

Supplemental Table S1

**
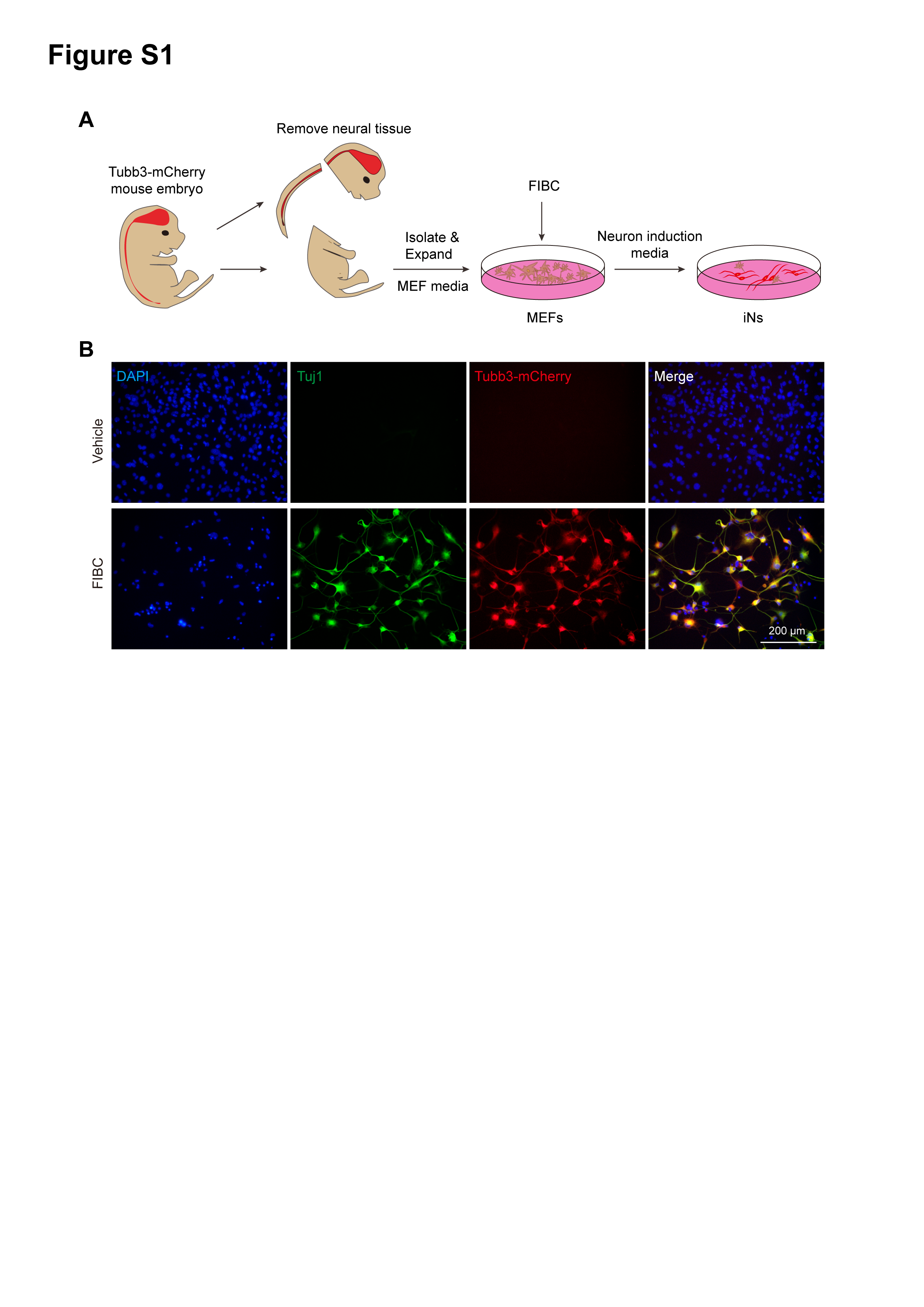
**

**Figure S1. Small molecules induced neuron reprogramming from MEFs.**

(A) Schematic illustration of experimental design for small molecule induced neuronal reprogramming from MEFs. MEFs, mouse embryonic fibroblasts; iNs, induced neurons; FIBC, Forskolin, ISX9, I-Bet, Chir99021.

(B) Immunofluorescent images of βIII tubulin expression (either by Tuj1 staining or Tubb3-mCherry reporter) and morphology of iNs from MEFs treated with either vehicle or FIBC.

**
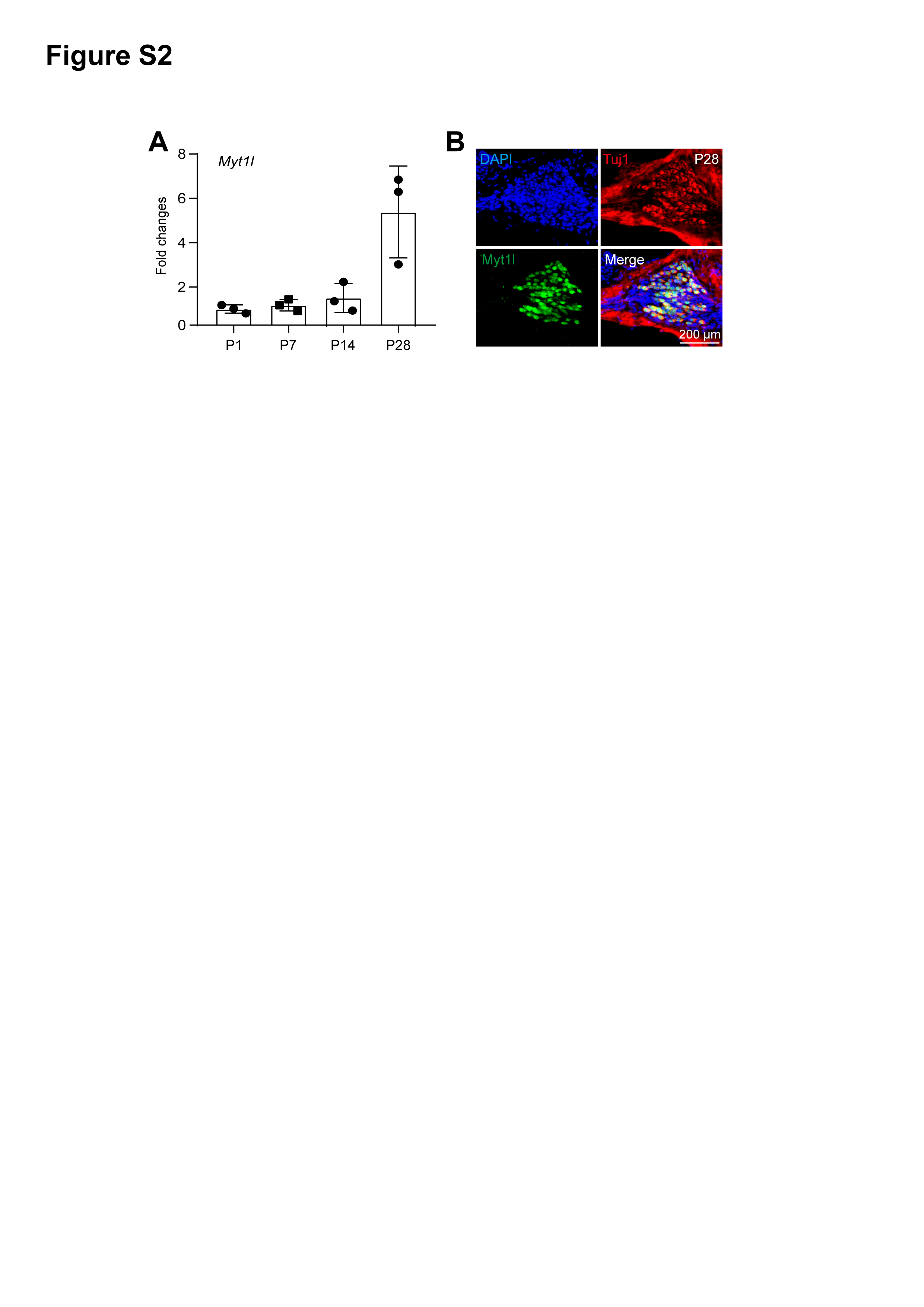
**

**Figure S2. Expression of Myt1l in mouse cochlea.**

(A) Temporal expression of Myt1l in postnatal whole cochlea. N = 3 cochleae at each age.

(B) Immunofluorescent images of Myt1l protein expression in spiral ganglion neurons from P28 mouse. Neurons were co-labeled with Tuj1.


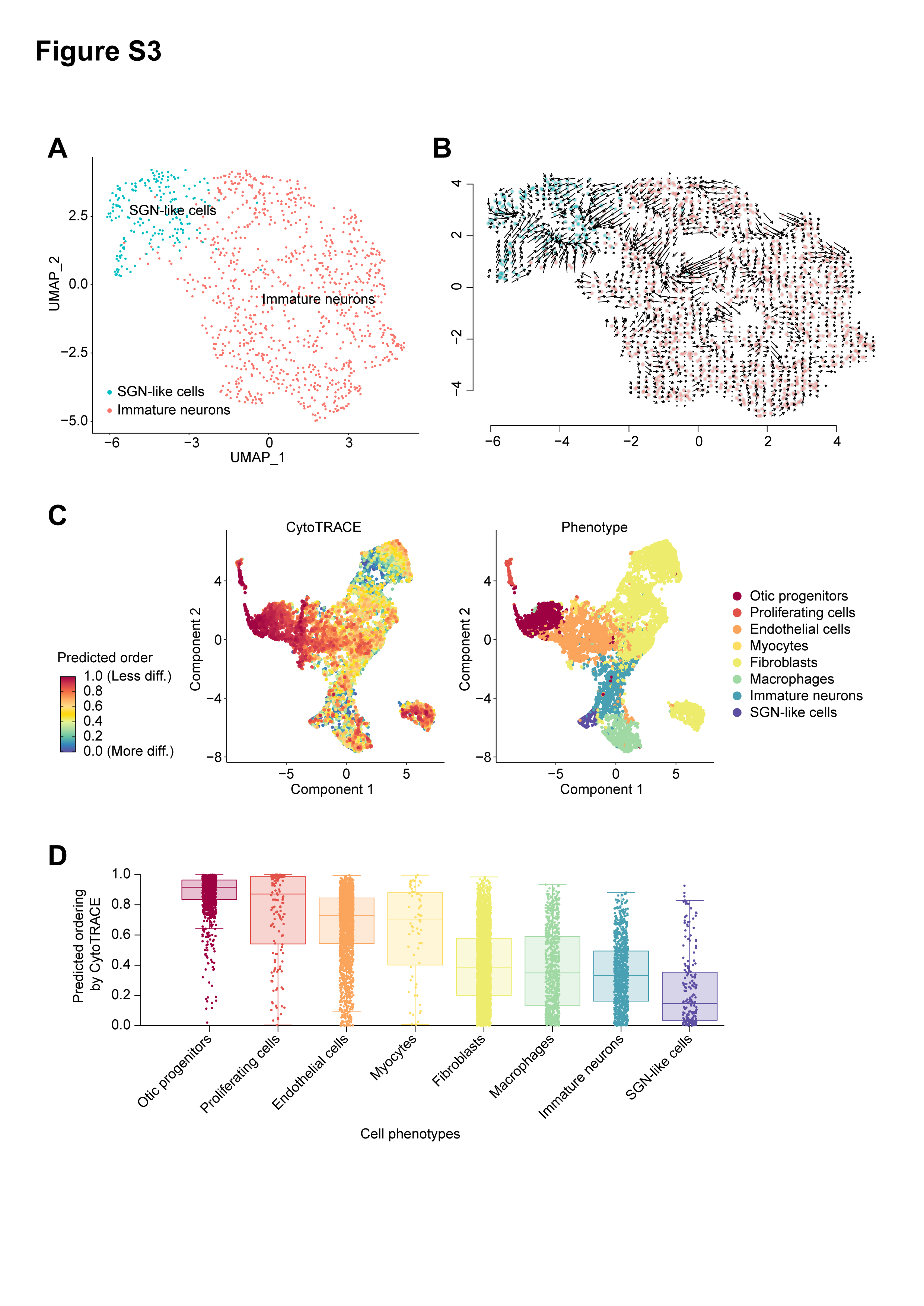


**Figure S3. RNA velocity and CytoTRACE analyses of the scRNA-seq results.**

(A) UMAP plots with SGN-like cells and immature neurons.

(B) RNA velocity analysis showing predicted cell fate transitions.

(C, D) CytoTRACE (A) heatmap and (B) bar chart showing the differentiation order of individual cell subpopulations.

**Table S1. Primers used for real-time qPCR**

| **Gene ID** | **Forward (5' to 3')** | | **Reverse (5' to 3')** |
| --- | --- | --- | --- |
| *Gapdh* | ACCACGAGAAATATGACAACTCAC | CCAAAGTTGTCATGGATGACC | |
| *Tubb3* | TAGACCCCAGCGGCAACTAT | GTTCCAGGTTCCAAGTCCACC | |
| *Nestin* | ACAGTGAGGCAGATGAGTTAGG | GAGGCAGGAGACTTCAGGTAG | |
| *Prox1* | TCTCAGCCAAACCCTCTC | CCGTTGACTGCGAATCTG | |
| *Sox2* | GCGGAGTGGAAACTTTTGTCC | CGGGAAGCGTGTACTTATCCTT | |
| *Slc17a7* | GTTCTGGCTTCTGGTGTCTTATG | CTCTCCAATGCTCTCCTCTATGT | |
| *Isl1* | CTTGCGGACCTGCTATGC | AACCACACTCGGATGACTCT | |
| *Scrt2* | GTCCTCTGCCTGTCCATTCCT | GCTGCCTCCCAAGTCTGTTC | |
| *Sox10* | CCAGGTGAAGACAGAGAC | AGACTGAGGGAGGTGTAG | |
| *Sox9* | GCAATACGACTACGCTGAC | ATGTAAGTGAAGGTGGAGTAGA | |
| *Pou3f4* | CTGGAGGAGGCTGATTCAT | GATGGAGGTTCGCTTCTTG | |
| *Pou4f1-endo** | AAACAAATAACCCACACCAAACAG | CTTCCTCAGAGCACCAGTTC | |
| *Ntrk3* | GTGACGAGCGAGGACAATG | GGTAGTAGACAGTGAGAGCAACA | |
| *S100a4* | TGGTCTGGTCTCAACGGTTA | TGGAAGGTGGACACAATTACATC | |
| *Neurog1* | GCTTCAGAAGACTTCACCTATGG | GTCGTGTGGAGCAGGTCTT | |
| *Neurog2* | GCACTTTATCGCCCGCTAG | CGCATAACGATGCTTCTCC | |
| *Neurod2* | CTGTCTCAACGGCAACTTC | GAGTAGTGCATAGAGTAGTGGTAG | |
| *Neurod4* | AGCATTAGTGGCAACTTCTC | GGTCTACAGGAACATCATAGC | |
| *Neurod6* | GCCTCAATGATGCTCTGGACAAT | CGGTCTCTTGCCAATCCTCAG | |
| *Isl2* | CCAACCCGCCTTTCAACAG | CGTCGCTACCGGAAGAGTT | |
| *Foxp1* | GCTTACTTCCGACGCAATG | TACTTCATCCACTGTCCATACTG | |
| *Nhlh1* | ATCCAGGAGTGGTCCAGGTC | CGCTCCTCACGACTCAAGT | |
| *Nhlh2* | GCAAACTACTACCCACGCT | GGTTGAGATAGGAGATGTAGCAGA | |
| *Klf4* | AGAACAGCCACCCACACTTG | TGCCTTGAGATGAGAACTCTTGGTA | |
| *Myc* | CTGCTCTCCATCCTATGTT | AAGTAACTCGGTCATCATCT | |
| *Hes6* | GGACGACCTGTGTTCTGAC | GCTGCCTAAGGATGTAGACA | |
| *Ascl3* | CCATGCCTTACACCAACTACA | CTCTCGCTCGTTCCTCTTG | |
| *Ascl2* | GCGTAAAGCTGGTAAACTTGG | GCGTCTCCACCTTACTCAG | |
| *Pou5f1* | TCACTCACATCGCCAATCA | CCTGTAGCCTCATACTCTTCTC | |
| *Pou4f1* | CTCGTCTGAGAAGATCGCCGCCATC | AAACCACACCCGCACCACGTTCTTT | |
| *Runx1* | CCAGGTAGCGAGATTCAAC | TCTATGGTAGGTGGCAACT | |
| *Pou3f4* | CTGGAGGAGGCTGATTCAT | GATGGAGGTTCGCTTCTTG | |
| *Atoh1* | CAGCAAACAGGTGAATGG | TTGAAGGACGGGATAACG | |
| *Atoh7* | ACAAGAAGCTGTCCAAGTACGAGAC | CGGCTAGGATGCGGGTGAG | |
| *Pou3f2* | CACCAGCATAGACAAGATC | CTTAGGGCATTTGAGGAAA | |
| *Pou4f2* | ACGCCACTTACCACACCAT | CGAGATGGGCACAGAAGAAGA | |
| *Pou4f3* | TCTGGCGGCGGTGGATAT | GCTGCTCATGGTATGGTAGGT | |
| *Rest* | CTGAGGGAGAGTTTGTGTGTAT | AAGTGGCGATTGAGGTGTT | |
| *Ptbp1* | AGAGGAGGCTGCCAACACTA | GTCCAGGGTCACTGGGTAGA | |
| *Prmt1* | GTGGATGGGTTACTGCCTCT | AAGCCATACACGTTCTCCCA | |
| *Myt1l* | CAGGAGTCAGCCACCCAACA | TCCAGCCGCATAAGGTTCAT | |
| *Ntrk2* | CAAAGCAATCGGGAGCATCT | CATCACCAGCAGGCAGAATC | |
| *Mafb* | TTCTTCCACCTCTTGCTACG | CTTGAACACCACCATTAAGTCTC | |
| *Ascl1* | AGATGAGCAAGGTGGAGACG | TGGAGTAGTTGGGGGAGATG | |
| *Ascl1-endo** | AAGAGTGACTGGTGTCTGAAC | GTGGTTGGGAGTTAATATAGTGTCT | |
| *Isl1-endo** | CTGAATGGTGCTGTTTCTATATTGG | CAAGTCACTGAGGCTGGTT | |
| *Prox1-endo** | AGACACAACTCCAGCATAC | CGTGAGCGATAGTTAGCAT | |
| *Scn8a* | GGACGAGCAGCCTGATTATG | CGATGATGACACCGATGAACA | |
| *Kcna2* | ATTGTATCTGTGATGGTCATTCTG | TGGTGCTGTTGGAATAGGT | |
| *Slc17a6* | TGCTACCTCACAGGAGAATGGA | GCGCACCTTCTTGCACAAAT | |
| *Grin1* | CCTTTCAGAGCACACTGTGGCT | CCAGGAAAACCACATGGCAGAG | |
| *Gria2* | TCTCTTCTAACAGCATACAGATAG | GCATAGACGCCTCTTGAA | |
| *Prph* | AGATAGCCACCTACAGGAAGC | TCTCAGGCACAGTCGTCTT | |
| *Syn1* | CCAATACAGGTTCTGCTATG | CACAAGTTCCACGATGAG | |
| *Snap25* | ATCTGGTGGCTCTAATTCCTAACTT | GACAGCACATTTGGAGAGATTCAG | |
| *Syp* | TGCCAACAAGACGGAGAG | GGAGTAGTCACCAACTAGGAAG | |
| *Map2* | GCCAGCCTCAGAACAAACAG | AAGGTCTTGGGAGGGAAGAAC | |
| *Gata3* | CCTCTCCTTCGGACCTCAC | GCTCTGCCTCTCTAACCCAT | |

*endo: endogenous transcript by targeting UTRs.
